# Evaluation of the Photocatalytic Activity of Distinctive-Shaped ZnO Nanocrystals Synthesized Using Latex of Different Plants Native to the Amazon Rainforest

**DOI:** 10.3390/nano12162889

**Published:** 2022-08-22

**Authors:** Robert S. Matos, John M. Attah-Baah, Michael D. S. Monteiro, Benilde F. O. Costa, Marcelo A. Mâcedo, Simone P. A. Da Paz, Rômulo S. Angélica, Tiago M. de Souza, Ştefan Ţălu, Rosane M. P. B. Oliveira, Nilson S. Ferreira

**Affiliations:** 1 Postgraduate Program in Materials Science and Engineering (P2CEM), Federal University of Sergipe, São Cristovão 49100-000, SE, Brazil; 2 Amazonian Materials Group, Federal University of Amapá (UNIFAP), Macapá 68911-477, AP, Brazil; 3 Laboratory of Corrosion and Nanotechnology (LCNT), Federal University of Sergipe, São Cristovão 49100-000, SE, Brazil; 4 University of Coimbra, CFisUC, Department of Physics, P-3004-516 Coimbra, Portugal; 5 Institute of Geosciences, Federal University of Pará, Belém 66075-110, PA, Brazil; 6 Núcleo de Engenharia de Materiais Sustentáveis (NEMaS), Universidade do Estado do Amapá, Macapá 68900-070, AP, Brazil; 7 The Directorate of Research, Development and Innovation Management (DMCDI), Technical University of Cluj-Napoca, 15 Constantin Daicoviciu St., 400020 Cluj-Napoca, Romania

**Keywords:** ZnO, green synthesis, Amapá latex, photocatalytic activity

## Abstract

ZnO nanocrystals with three different morphologies have been synthesized via a simple sol-gel-based method using *Brosimum parinarioides* (bitter Amapá) and *Parahancornia amapa* (sweet Amapá) latex as chelating agents. X-ray diffraction (XRD) and electron diffraction patterns (SAED) patterns showed the ZnO nanocrystals were a pure hexagonal wurtzite phase of ZnO. XRD-based spherical harmonics predictions and HRTEM images depicted that the nanocrystallites constitute pitanga-like (~15.8 nm), teetotum-like (~16.8 nm), and cambuci-like (~22.2 nm) shapes for the samples synthesized using bitter Amapá, sweet Amapá, and bitter/sweet Amapá chelating agent, respectively. The band gap luminescence was observed at ~2.67–2.79 eV along with several structural defect-related, blue emissions at 468–474 nm (V_O_, V_Zn_, Zn_i_), green emissions positioned at 513.89–515.89 (h-$V_{O}^{+}$), and orange emission at 600.78 nm ($V_{O}^{+}$–$V_{O}^{++}$). The best MB dye removal efficiency (85%) was mainly ascribed to the unique shape and oxygen vacancy defects found in the teetotum-like ZnO nanocrystals. Thus, the bitter Amapá and sweet Amapá latex are effective chelating agents for synthesizing distinctive-shaped ZnO nanocrystals with highly defective and remarkable photocatalytic activity.

## 1. Introduction

The morphology-controlled synthesis of metal oxide nanocrystals has gained much scientific interest because of their modulated physical properties induced by intrinsic defects associated with different morphologies [1,2]. Among them, ZnO has attracted more attention for its extensive applications in photocatalysts, ultraviolet blocks, and dye-sensitized solar cells [3,4,5,6]. ZnO is a semiconductor whose crystalline lattice can be easily engineered with different structural defects during crystal growth [7,8]. The manipulation of the structural and morphological aspects of ZnO nanocrystals has generated promising results that have expanded its study and applications [9]. However, it is known that traditional physical and chemical processes for obtaining ZnO nanocrystals are costly and unsafe.

Recently, substantial effort has also been directed toward the green synthesis of ZnO nanocrystals using sustainable and environmentally friendly methods without expensive, harsh, and toxic chelating. Recent reports have proved several different plant extracts to be efficient chelating or complexing agents to synthesize nanocrystals [10,11]. However, not much is reported on using plants’ latex for synthesizing controlled size and shape ZnO nanocrystals specifically. In this regard, Brosimum parinariodides Ducke (sweet Amapá) and *Parahancornia amapa* Ducke (bitter Amapá) are Amazon rainforest plants that are known for their complex polymeric matrices latex composition [12,13]. The abundance of organic compounds in the Amapá latex has a favorable chemical environment for the chelation of Zn^2+^ ions and the subsequent formation of ZnO crystals. Nonetheless, these substances have only been commonly used to treat diseases in the traditional Amazon rainforest communities [14].

Several other recent reports have also demonstrated that green synthesized ZnO nanocrystals can be used for photocatalytic purposes [15,16,17,18,19]. Such approaches have been increased due to the expansion of the textile industry, where factories use synthetic dyes composed of $R-N=N-R^{\prime }$ chromophore group that is generally part of aromatic rings [20]. This growing demand has been justified because, in 2021, its use moved around USD 32.9 billion [21] and is projected to reach USD ~49 billion by 2027 [22]. The dyes are water soluble and stable compounds that contaminate billions of liters of wastewater worldwide. Currently, it is estimated that between 17 and 20% of the wastewater is contaminated by dyes from the textile industry [23,24]. Thus, eco-friendly approaches for obtaining ZnO crystals can be considered a sustainable and safe way to fabricate nanocrystals that can be used to purify dye-contaminated wastewater.

Herein, we aim to complete the limited literature on synthesized ZnO using plant latex, reporting for the first time the green synthesis of highly crystalline, single-phase, and distinctive-shaped ZnO nanocrystals using sweet Amapá and bitter Amapá latex as effective chelating agents. The impact of these different chelating agents on the purity, size, shape, and optical nature of the ZnO nanocrystal was investigated. Additionally, a typical photocatalytic experiment was performed to evaluate the removal efficiency of methylene blue (MB) dye, a known azo toxic and stable pollutant.

## 2. Materials and Methods

### 2.1. Synthesis

This study selected two Amazon rainforest plants, *Brosimum parinarioides* (bitter Amapá) and *Parahancornia amapa* (sweet Amapá). The sweet Amapá (SA) and bitter Amapá (BA) latex samples were collected in Vitória do Jari, Amapá, Brazil, at 0°52′25″ S and 52°24′06″ W (SA) and 0°57′08″ S and 52°24′45″ W (BA). In a typical experiment, 1 g of Zn(NO_3_)_2_·6H_2_O (Sigma-Aldrich, St. Louis, MO, USA, 99.99%) was dissolved in 10 mL (BA), 10 mL (SA), and 10 mL (5 mL of BA + 5 mL of SA, called SBA) of Amapá latex and stirred for 10 min aimed at obtaining solutions with a concentration of 100 g·L^−1^. Afterward, the solutions were maintained at 100 °C for 24 h, and the obtained xerogels were calcinated at 400 °C. The calcined samples indicated high productivity of ZnO nanocrystals with yields of about 92% (BA), 98% (SA), and 85% (SBA).

### 2.2. Characterization

The formation of ZnO nanocrystals was confirmed using thermogravimetric and differential thermal analyses (TGA/DTG, NETZSCH-STA-449F3 Jupiter). Their functional groups were obtained by a Fourier transform infrared spectrometer (FTIR) operating 4000–400 cm^−1^ on a Bruker Vertex 70 spectrophotometer. The structural characteristics of the nanocrystals were investigated by X-ray diffraction (XRD) measurements that were performed by using an Empyrean, PANalytical X-ray diffractometer with Co-Kα radiation (λ = 1.78901 Å), in the range of 30°–90° (2θ) range; 40 kV and 40 mA; 0.01° (2θ) step size and 20 s per step. The instrument resolution was assessed using the LaB6 NIST/SRM 660b standard. Rietveld refinement was performed using the FullProf software [25]. The morphological analysis as well as electron diffraction patterns (SAED), were obtained by transmission electron microscopy (TEM) using a Tecnai G2-F20 S-TWIN microscope operating at 200 kV. The photoluminescence measurements were performed using a fluorescence spectrophotometer (Jasco, FP-8600) operating at 200–800 nm.

### 2.3. Photocatalytic Tests

In a typical photocatalytic experiment, 10 mg of ZnO nanocrystals were suspended in a stable methylene blue (MB) solution (10 mg·L^−1^). The resultant suspension was kept under a stirrer and irradiated using a visible light source (600-W halogen lamp) with an irradiance of 7.74 W·m^−2^ for 120 min. Before this, a similar experiment was performed in the dark as a control experiment. The MB band reduction at λ_max_ = 664 nm was evaluated in a UV–vis spectrophotometer (VARIAN, Cary 100), using 5 mL aliquots collected at intervals of 20 min. The MB dye removal efficiency was determined using a relation between the absorption at 664 nm and equilibrium concentration before (*A*_0_ and *C*_0_) and after (*A* and *C*) irradiation, as represented in Equation (1).

(1) $$\%\;\mathrm{Removal}\;\mathrm{efficiency}=\left(\frac{C_{0}-C}{C_{0}}\right)\times 100\%=\left(\frac{A_{0}-C}{A_{0}}\right)\times 100\%$$

The photodegradation reaction kinetic was estimated using a Langmuir–Hinshelwood kinetic model according to Equation (2).

(2) $$kt=-ln\left(\frac{C}{C_{0}}\right)$$

where *k* is the pseudo-first-order rate constant, and *t* is the irradiation time.

## 3. Results and Discussion

**Figure 1:**
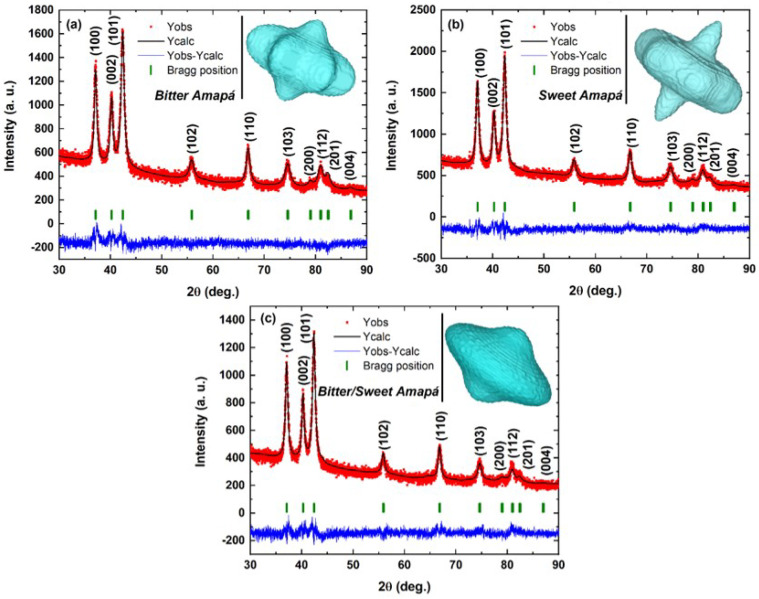
XRD measurements and calculated patterns for ZnO nanocrystals synthesized using: (**a**) bitter Amapá, (**b**) sweet Amapá, and (**c**) bitter/sweet Amapá. The insets show the respective 3D representation of the crystallite shape predicted via an SPH approach.

The successful synthesis was confirmed by the high yield (85–98%) productivity of ZnO nanocrystals. Furthermore, the purity and quality of the produced ZnO nanocrystals were confirmed by characterizations performed using different techniques. Figure 1 shows the observed, calculated, and different XRD profiles for ZnO nanocrystals synthesized with different Amazon rainforest latex chelating agents. The peaks could be indexed to the hexagonal structure of ZnO, belonging to the P63mc space group (ICSD#76641), without secondary phases. The crystalline domain shape and size estimated from XRD data by an SPH approach in parallel to the Rietveld refinement [4] predicted pitanga-like (D_XRD_ = 15.8(4) nm (BA), teetotum-like (D_XRD_ = 16.8(3) nm) (SA), and cambuci-like (D_XRD_ = 22.2(5) nm) (SBA) shapes, as shown in 3D representation in the insets of Figure 1.

**Figure 2:**
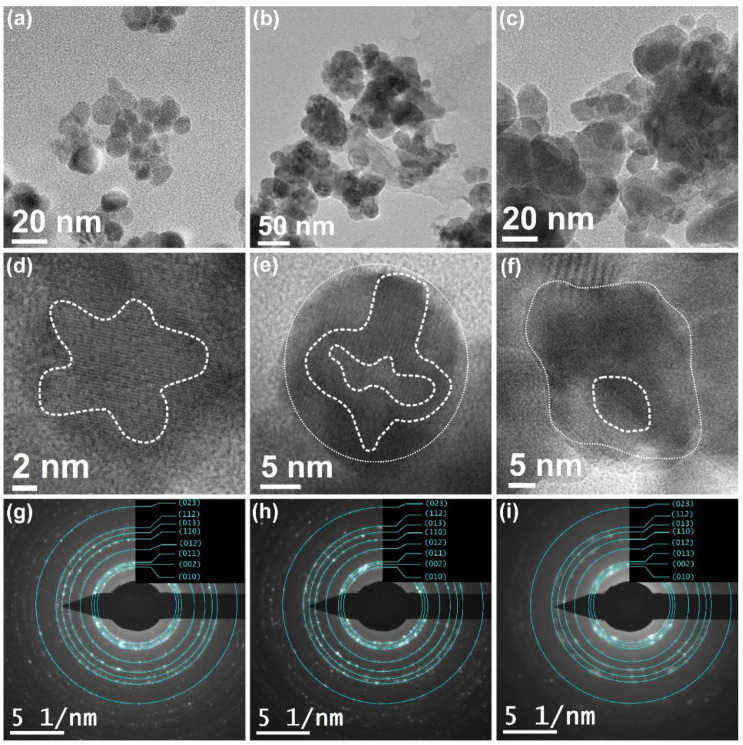
TEM, HRTEM micrographs, and SAED of ZnO nanocrystals, respectively, synthesized using: (**a**,**d**,**g**) bitter Amapá, (**b**,**e**,**h**) sweet Amapá, and (**c**,**f**,**i**) bitter/sweet Amapá.

**Figure 3:**
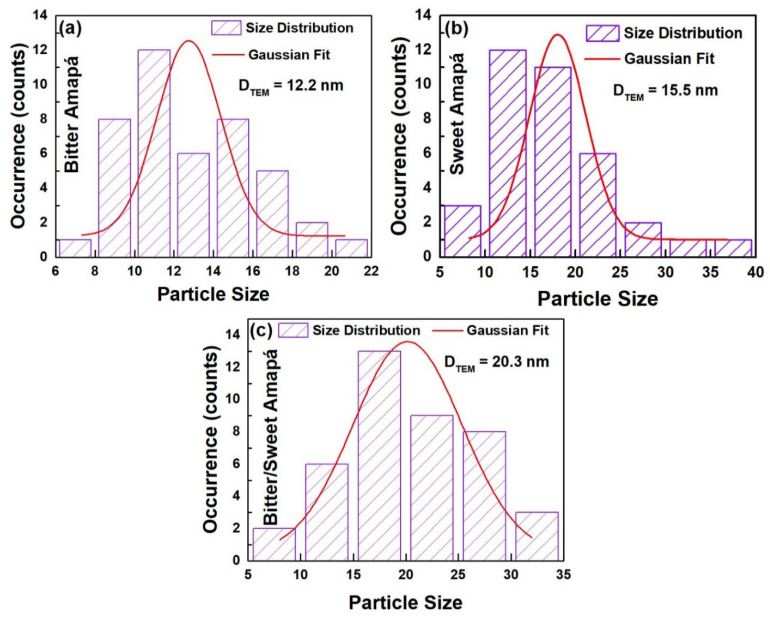
Particle size distribution was obtained from TEM images of (**a**) pitanga-like, (**b**) teetotum-like, and (**c**) cambuci-like ZnO nanocrystals.

The TEM analysis consistently showed the reliability of the XRD-based crystal shape predicted results, as shown in Figure 2. The TEM images (Figure 2a–c) and Figure S1 in Supplementary Materials clearly show agglomerated nanocrystals, whereas average particle sizes for the individual powders ranged from 12.2–20.3 nm (Figure 3). Furthermore, high-resolution TEM (HRTEM) shown in Figure 2d–f confirmed the pitanga-like, teetotum-like, and cambuci-like morphology of ZnO nanocrystals, approving the consistency of the XRD-based SPH predicted shapes. The SAED image (Figure 2g–i) shows concentric rings corresponding to the planes (100), (002), (101), (102), (110), and (103) of a wurtzite ZnO phase, corroborating the XRD results.

**Figure 4:**
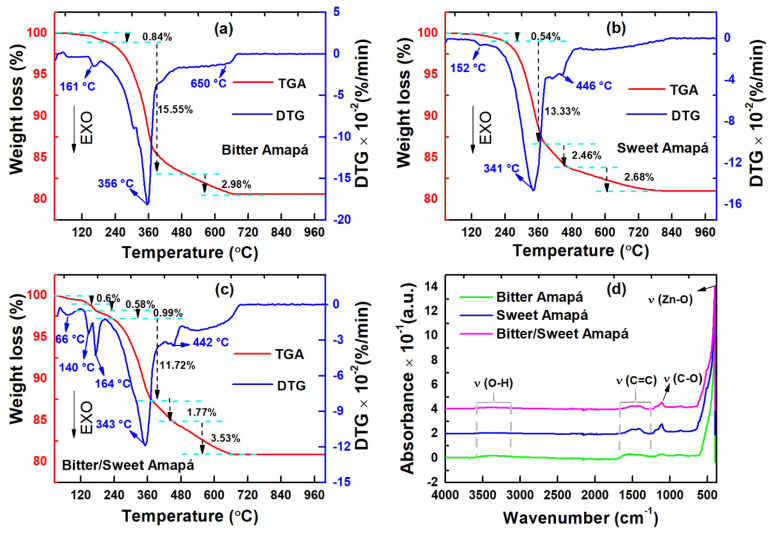
(**a**–**c**) TGA/DTG curves of the obtained xerogels and (**d**) FTIR spectra of the ZnO nanocrystals calcined at 400 °C.

Figure 4a–c shows TGA/DTG curves of ZnO xerogels obtained using BA, SA, and SBA as chelating agents. Weight losses of 0.84% and 0.54%, accompanied by exothermic peaks at 161 °C and 152 °C, are observed for the BA (Figure 4a) and SA (Figure 4b) samples, respectively. Meanwhile, the SBA sample (Figure 4c) presented three exothermic peaks at 66, 140, and 164 °C, which is associated with a weight loss of 2.15%. These weight losses were ascribed to the elimination of water and decomposition of ${\mathrm{NO}}_{3}^{-}$ in the xerogels. For all samples, the higher weight loss that occurred between 250 and 400 °C (11.72–15.55%) is attributed to the latex release of CO_2_ and other volatile compounds. Prominent exothermic peaks at 356 (BA), 341 (SA), and 343 °C (SBA) are associated with ZnO crystallization. Additionally, ~5% (weak peaks above 400 °C) weight losses after crystallization are attributed to the release of CO_2_ from residual latex. These results confirm that 400 °C is assigned as a suitable calcination temperature to obtain stable ZnO nanocrystals.

The functional groups present in freeze-dried BA and SA latex, as well as those adsorbed on the surface of the ZnO nanocrystals, were investigated by FTIR, as shown in Figure S2 and Figure 4d, respectively. The BA and SA latex FTIR spectra reveal the presence of several organic compounds (Figure S2). The band centered between 3050 and 3712 cm^−1^ is attributed to the stretching vibration of hydroxyl (O−H) and amines (N−H) groups. The bands positioned at 2877−3056 cm^−1^ and 2800−2877 cm^−1^ are due to stretching vibration of C−H related to alkenes and alkanes, respectively. These vibrations are explicitly linked to the presence of −CH_3_ and −CH_2_, probably due to lipids and proteins [4,26] of the latex, which is common in plant extracts. The bands located at 1738, 1720, 1670, and 1650 cm^−1^ are associated with the carbonyl group and arise as a result of the existence of ester, aldehydes, carboxylic acids, and amides functional groups, respectively. Moreover, bands related to axial angular deformation of C=C are also observed between 1417 and 1584 cm^−1^. However, a C=C stretch vibration is only observed for BA latex. The band located at 1250 cm^−1^ is related to the C−N stretching vibration in the rings, probably associated with alkaloids and proteins present in the latexes. Further, the bands centered at 1180, 1080, and 1040 cm^−1^ are due to the C−O stretching vibration of esters, carboxylic acids, and secondary alcohol, respectively, probably as a consequence of the presence of mixed chemical structures such as carbohydrates. Finally, the aromatic rings are characterized by weak bands positioned around 880, 825, and 725 cm^−1^. These bands can be attributed to angular deformation of the aryl group (δ_ArH_), O−H, and C−H present in the aromatic rings of volatile compounds of the latex. On the other hand, the nanocrystal’s FTIR spectra reveal a highly significant disappearance of the bands referring to the organic compounds (Figure 4d). A weak O−H stretching vibration associated with water adsorbed on the surface of the nanocrystals is observed around 3455 cm^−1^ in all samples. The stretching vibrations of the C=C and C−O bonds at 1475 and 1122 cm^−1^ are associated with organic compounds not eliminated during calcination [27]. This is in complete agreement with the TGA/DTG analysis because after 400 °C an elimination of some residual organic compounds was observed. Finally, the band recorded at ~412 cm^−1^ is characteristic of Zn−O stretching vibration [28]. The XRD findings and TEM images well support such results.

Previous studies have shown that BA is rich in organic acids, alkaloids, anthraquinones, depsides, and depsidones, while SA contains alkaloids, coumarin derivatives, steroids, triterpenoids, and purines [12,13,29]. As can be seen, alkaloids, which are aromatic substances, represent the common components for both latexes. However, BA must have greater amounts of alkaloids because this milk has a strong and bitter taste and a unique aroma, which often gives the repellent action [13]. The chemical groups shown in the FTIR spectra are found in these compounds. For instance, Salles [12] isolated five important steroids butyrospermol, tirucalla-7,24-dien-3β-ol, cycloartenol, cycloeucalenol, and obtusifoliol from the dichloromethane extract of the latex of SA, where the presence of secondary alcohol and alkenes in the chain of all compounds is observed. This confirms the presence of these organic groups in this latex, mainly because the secondary alcohol found is formed in the steroid rings and the O−H band positioned at 825 cm^−1^ was computed only for the SA. Using extracts from the bark of sweet Amapá, Sá et al. [30] isolated four flavonoids, parinarioidine A and B, licograchalcone, and kanzonol from bark extract of SA, where the presence of other chemical functions such as ether, phenol, and ketone can be confirmed. Palheta et al. [31] also show that the rich physicochemical composition of SA includes lipids, proteins, ash, carbohydrates, and fibers. On the other hand, Sobrinho et al. [32] and Carvalho [33] isolated mixtures of hydroxylated and non-hydroxylated lupeol esters from latex and roots of BA, respectively, revealing a persistent presence of esters. Carvalho et al. [34] also registered the presence of secondary alcohol in pentacyclic triterpenes of the BA. Thus, bitter and sweet Amapá latex comprise the most common organic constituents, such as α-and β-amyrin and lupeol acetates. However, there are also distinct constituents, such as a mixture of hydroxylated esters (BA), cycloeucalenol, and obtusifoliol (SA). These organic constituents most likely dictate the size and shape evolution of the ZnO nanocrystals at different reaction stages. Initially, hydroxyl groups in α-and β-amyrin acetates have a stronger ability to bind divalent Zn^2+^ cations. Afterward, lupeol acetate increases the solution viscosity by decreasing mass transfer to stabilize the medium by growth $\mathrm{Zn}{\left(\mathrm{OH}\right)}_{4}^{2-}$ units. Thus, the interaction of $\mathrm{Zn}{\left(\mathrm{OH}\right)}_{4}^{2-}$ units with hydroxylated esters (BA), cycloeucalenol, and obtusifoliol (SA), leading to straightforward anisotropic growth of ZnO nanocrystals. It is suggested that hydroxylated esters would induce changes in the growth rate of different crystal faces, resulting in the formation of pitanga-like structures. However, cycloeucalenol and obtusifoliol are most likely to induce a common crystal face sharing to decrease the high surface energy by maximizing the packing density resulting in the formation of teetotum-like structures. Nevertheless, as BA:SA ratio was 1:1, a slow rate of isotropic growth occurred from nucleating aggregated seeds without a specific direction, leading to the thermodynamically favored change of the nanocrystal shape to cambuci-like morphology.

**Figure 5:**
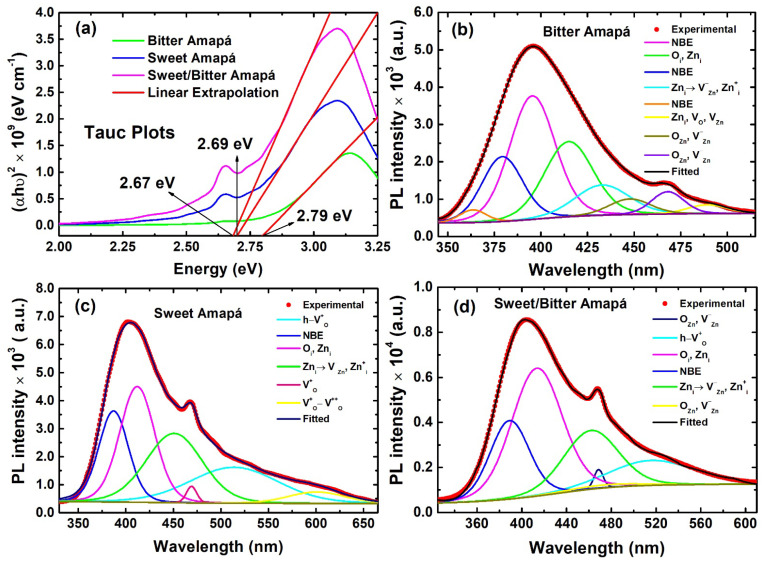
(**a**) Tauc plots and (**b**–**d**) deconvoluted PL spectra of the ZnO nanocrystals calcinated at 400 °C.

The optical properties of ZnO nanocrystals were demonstrated via photoluminescence (PL) using a λ_exc_ = 250 nm at room temperature. The spectra were used to estimate the optical bandgap of the samples (Figure 5a), whose values are 2.67 eV (SA), 2.69 eV (BA), and 2.79 eV (SBA). These values are significantly lower than reported for ZnO bulk (~3.3 eV) [4], suggesting that different carbon impurities adsorbed on the nanocrystal surface act as a capping agent, which is usually observed in green synthesized ZnO nanocrystals [35]. The fitted PL (Figure 5b,c) spectra reveal that ZnO band-edge peaks are in different but close positions at ~396 nm (BA), 405 nm (SA), and 404 nm (SBA). Moreover, UV emissions at 363.49, 379.28, and 395.44 nm (BA), 387 nm (SA), and 398.11 nm (SBA) are ascribed to near-band-edge emission in ZnO nanocrystals [4]. Violet emissions at 414.93 nm (BA), 411.97 nm (SA), and 413.49 nm (SBA) are associated with oxygen interstitial (O_i_) [36] or zinc interstitials (Zn_i_) [4]. Blue emissions at 432.78 nm and 447.7 nm are assigned to transitions from Zn_i_ to neutral zinc vacancies ($V_{\mathrm{Zn}}^{-}$) [37] and singly ionized zinc interstitials (${\mathrm{Zn}}_{i}^{+}$) [4] in all samples. Additionally, a blue transition at 450 nm is associated with singly ionized oxygen vacancies ($V_{O}^{+}$) [38] is present only for SA sample. However, blue emissions at 468 nm (BA), 469 nm (SA), and 461, 468, and 474 nm (SBA) arise due to the oxygen antisites (O_Zn_) and negatively charged zinc vacancies ($V_{\mathrm{Zn}}^{-}$) [4]. Further, at 489.31 nm, a blue emission associated with trap state transition can be caused by neutral oxygen vacancies (V_O_), V_Zn_, and Zn_i_ along with the crystal [39] is observed in the BA sample. The green emissions ranging from 513.89–515.89 nm for the SA and SBA samples are ascribed to the recombination of a photogenerated hole with a $V_{O}^{+}$ state [38]. The orange emission at 600.78 nm only for the SA sample emerges due to deep donor defects, where a hole trapped by $V_{O}^{+}$ from valence band provide a new double ionized oxygen vacancy $V_{O}^{++}$ state [40].

**Figure 6:**
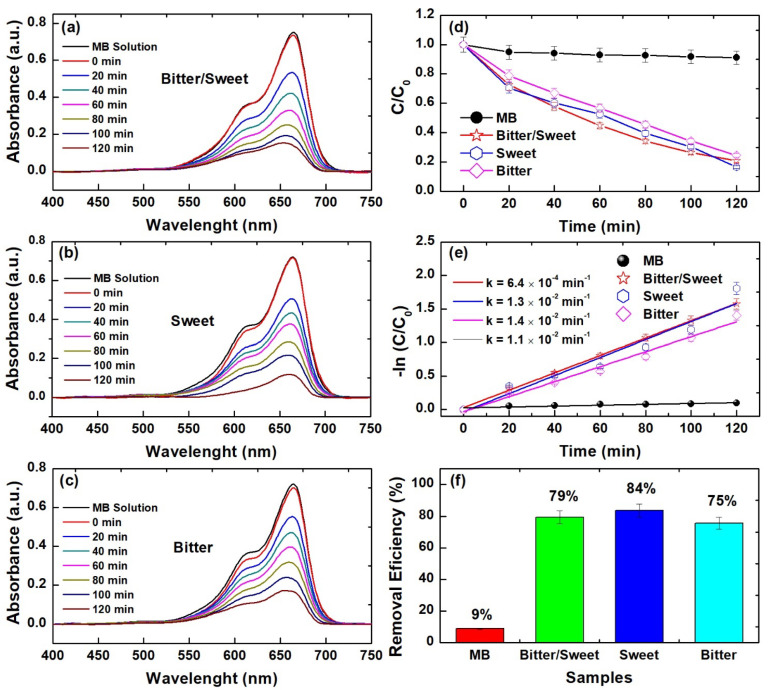
(**a**–**c**) Time-dependent UV–vis spectra, (**d**) C/C_0_ ratio, (**e**) first-order discoloration kinetics, and (**f**) removal efficiency of visible light-mediated M.B. Dye degradation in solutions containing pitanga-like, teetotum-like, and cambuci-like ZnO nanocrystals.

The defective distinctive-shaped ZnO nanocrystals were tested as photocatalysts using a typical photocatalytic experiment, whose results are shown in Figure 6. As can be seen, for all experiments, the MB dye band at 664 nm displays a robust reduction after 120 min of irradiation with visible light (Figure 6a–c). However, the most pronounced reduction was observed in the experiment containing teetotum-like ZnO nanocrystals (Figure 6b). Despite that, a small difference is observed between the C/C_0_ ratios of the samples after 120 min, although it has been computed as a remarkable reduction compared to the experiment in the dark (MB). Moreover, the samples exhibited similar photocatalytic reaction rates, but the highest value was recorded for the solution containing teetotum-like ZnO nanocrystals (1.4 × 10^−2^ min^−1^). The highest overall removal efficiency was also observed for the sample with teetotum-like ZnO nanocrystals (84%), which is statistically similar to the sample containing cambuci-like ZnO nanocrystals (79%). Comparatively, our teetotum-like ZnO nanocrystals are more efficient and degrade MB molecules faster than the near-spherical activated by $\left[V_{\mathrm{Zn}}+V_{O}^{+}\right]$ defects reported by Ferreira et al. [4] that found the maximum degradation of 30.67% and reaction rate of 2.9 × 10^−3^ min^−1^ after 120 min of irradiation with visible light. The defective spherical nanocrystals of Aldeen et al. [18] displayed an MB overall degradation of ~98%, but the reaction kinetic (3.7 × 10^−3^ min^−1^) was significantly slower than the reported in the present work. Moreover, these authors have used UV illumination, which is a more energetic light source and properly can lead to the destruction of MB molecules. The green synthesized ZnO nanorods obtained by [41] could degrade up to 97.32% of the MB organic molecules at a reaction speed of 1.94 × 10^−2^ min^−1^, which is faster than 1.4 × 10^−2^ min^−2^ found in our work. However, the high exposure time of 150 min used by these authors suggests that if our exposure time is increased, the overall degradation can reach 100%. These literature observations prove that the nanocrystals’ shape combined with their structural defects plays a critical role in the degradation of MB dye molecules in an aqueous solution, confirming that our defective distinctive-shaped nanocrystals are effective for the destruction of MB dye molecules.

Literally, the photocatalytic performance of ZnO nanocrystals has been associated with their shape, particle size, surface contamination, and structural defects [4,42]. The FTIR analysis (Figure 4d) showed that there is similar surface contamination in all nanocrystals, suggesting that the impurities adsorbed on the surface of the nanocrystals are not expected to influence the photocatalytic process. However, our structural (Figure 2) and morphological (Figure 3) evaluations clearly showed that the nanocrystals have different particle sizes and shapes. Hence, the best removal efficiency of the SA sample is ascribed to its unique 15.5 nm teetotum-like nanocrystals. Additionally, the PL analysis revealed that ZnO nanocrystals have a variety of native structural defects, but the coexistence of (h-$V_{O}^{+}$) and ($V_{O}^{+}$–$V_{O}^{++}$) was only recorded for the SA sample. This occurred because during the growth of teetotum-like ZnO nanocrystals, the ${\mathrm{Zn}}^{2+}$ species migrated to interstitial and $O^{2-}$ positions in order to stabilize the ZnO lattice, creating both $V_{\mathrm{Zn}}$ and ${\mathrm{Zn}}_{i}$ defects. Furthermore, the oxygen also migrated to interstitial positions promoting the formation of $O_{i}$ and $V_{O}$, that aids to complete crystal stabilization underwent two ionization processes, thus, releasing electrons to nanocrystals’ surface and favoring the formation of $V_{O}^{+}$ and $V_{O}^{++}$ defects. Thus, the teetotum-like shape combined with the symbiotic action of oxygen vacancies is responsible for the enhanced photocatalytic performance of the SA sample.

**Figure 7:**
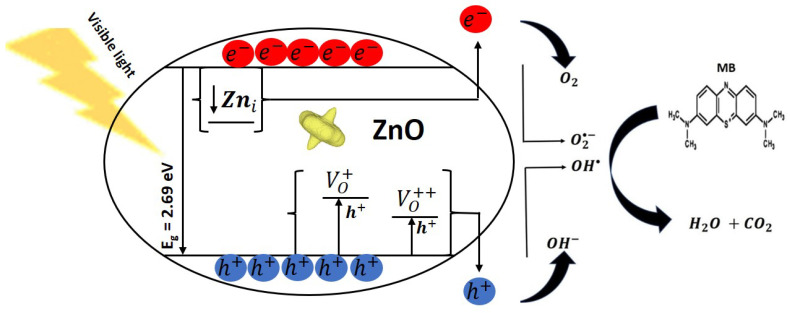
Mechanism of the photocatalytic reaction of the degradation of MB dye molecules using teetotum-like ZnO nanocrystals synthesized using sweet Amapá milk as a chelating agent.

The overall mechanism responsible for the photocatalytic degradation of MB dye in aqueous solutions containing teetotum-like ZnO nanocrystals is represented in Figure 7 and was elaborated as follows. After the absorption of light by the nanocrystals (E ≥ 2.69 eV), the electrons from the valence band (VB) migrate to the conduction band (CB), generating charge carriers that are transported to the surface of the nanocrystals Equation (3). On the surface, $e_{\mathrm{CB}}^{-}$ and $h_{\mathrm{VB}}^{+}$ interact with oxygen $O_{2}$ and water molecules $H_{2}O$ to form superoxide $O_{2}^{•-}$ (Equation (4)) and hydroxyl radicals ${\mathrm{OH}}^{•}$ (Equation (5)), where $O_{2}^{•-}$ is further used to production of more free ${\mathrm{HO}}^{•}$ groups that increase the destruction of MB organic molecules [4]. The reactions described above are also valid for the pitanga-like and cambuci-like nanocrystals. However, the enhanced photocatalytic behavior of teetotum-like ZnO nanocrystals was attributed to its unique shape and oxygen vacancy defects. These defects trap holes that delay the recombination of charge carriers, where such holes are used on the nanocrystals’ surface for the generation of ${\mathrm{HO}}^{•}$ and hydrogen peroxide (H_2_O_2_) molecules (Equations (6) and (7)). H_2_O_2_ molecules are easily photocatalyzed to generate ${\mathrm{OH}}^{•}$ species [43] (Equation (8)).

(3) $$\mathrm{Teetotum}-\mathrm{like}\;\mathrm{ZnO}\;\mathrm{nanocrystals}+\mathrm{hv}\;\to \left(e_{\mathrm{CB}}^{-}\right)+\left(h_{\mathrm{VB}}^{+}\right)$$

(4) $$O_{2}+e_{\mathrm{CB}}^{-}\to O_{2}^{•-}$$

(5) $$H_{2}O+h_{\mathrm{VB}}^{+}\to H^{+}+{\;\mathrm{HO}}^{•}$$

(6) $$h_{h-V_{O}^{+}}^{+}+O_{2}^{•-}+2H_{2}O\to 2H^{+}+2{\mathrm{HO}}^{•}+H_{2}O_{2}$$

(7) $$h_{V_{O}^{+}-V_{O}^{++}}^{+}+O_{2}^{•-}+2H_{2}O\to 2H^{+}+2{\mathrm{HO}}^{•}+H_{2}O_{2}$$

(8) $$H_{2}O_{2}+\mathrm{Visible}\;\mathrm{Light}\to 2{\mathrm{HO}}^{•}$$

The chemical species $O_{2}^{•-}$ and ${\mathrm{HO}}^{•}$ interact with the MB organic molecules leading to their destruction, where the final byproducts are expected to be $H_{2}{O\;\mathrm{and}\;\mathrm{CO}}_{2}$ molecules [44,45]. Therefore, our ZnO nanocrystals were effective for the destruction of MB dye molecules in an aqueous solution under visible light. The singular shape further improved this destruction and structural defects found in the ZnO nanocrystals synthesized with sweet Amapá milk.

## 4. Conclusions

In summary, single-phase ZnO nanocrystals of different sizes and shapes have been synthesized using different Amazon rainforest plant latex chelating agents. Using bitter Amapá, sweet Amapá, and bitter/sweet Amapá latex is critical for forming pitanga-like (~15.8 nm), teetotum-like (~16.8 nm), and cambuci-like (~22.2 nm) shaped ZnO nanocrystals, respectively. These morphologies favored the existence of several structural defects such as O_i_, Zn_i_, Zn_i_→${\mathrm{Zn}}_{i}^{+}$, $V_{O}^{+}$, O_Zn_, $V_{\mathrm{Zn}}^{-}$, V_O_, V_Zn_, and $V_{O}^{++}$. Our findings prove that bitter and sweet Amapá latex are effective chelating agents. The discoloration of the MB dye in aqueous solutions containing the nanoparticulate synthesized with bitter, sweet, and bitter/sweet latex was determined to be 79%, 84%, and 75%, where the best performance was attributed to the coexistence of different ionized oxygen vacancies defects found in the teetotum—like ZnO nanocrystals. Our results proved that using bitter Amapá, sweet Amapá, and bitter/sweet Amapá latex as chelating agents for synthesizing distinctive-shaped ZnO nanocrystals gives rise to a promising eco-friendly way for the obtention of nanocrystals with suitable physical properties for application in photocatalysis.

## Data Availability

The data that supports the findings of this study are available from the corresponding author upon reasonable request.

## Supplementary Materials

The following supporting information can be downloaded at: https://www.mdpi.com/article/10.3390/nano12162889/s1, Figure S1: TEM images of (a) pitanga-like, (b) teetotum-like, and (c) cambuci-like ZnO nanoparticles, Figure S2: FTIR Spectra of freeze dried Bitter-Amapá (*Parahancornia amapa* Ducke) and Sweet-Amapá (*Brosimum parinariodides* Ducke) Amazon rainforest latex.
