# Supplementary material for: Evaluation of the Photocatalytic Activity of Distinctive-Shaped ZnO Nanocrystals Synthesized Using Latex of Different Plants Native to the Amazon Rainforest
Source: Nanomaterials (Basel). 2022 Aug 22;12(16):2889. doi: 10.3390/nano12162889 (PMC9416145; doi:10.3390/nano12162889)
Supplement: Supplementary file 1 [file nanomaterials-12-02889-s001.zip › nanomaterials-1877068-supplementary.pdf]

nanomaterials

Supplementary Materials

Evaluation of the Photocatalytic Activity of Distinctive-Shaped ZnO Nanocrystals Synthesized Using Latex of Different Plants Native to the Amazon Rainforest

Robert S. Matos [1,2], John M. Attah-Baah [3], Michael D. S. Monteiro [3], Benilde F. O. Costa [4], Marcelo A. Mâcedo [3], Simone P. A. Da Paz [5], Rômulo S. Angélica [5], Tiago M. de Souza [6], Ştefan Ţălu [7,*], Rosane M. P. B. Oliveira [1] and Nilson S. Ferreira [3,*]

[1] Postgraduate Program in Materials Science and Engineering (P²CEM), Federal University of Sergipe, São Cristovão 49100-000, SE, Brazil
[2] Amazonian Materials Group, Federal University of Amapá (UNIFAP), Macapá 68911-477, AP, Brazil
[3] Laboratory of Corrosion and Nanotechnology (LCNT), Federal University of Sergipe, São Cristovão 49100-000, SE, Brazil
[4] CFisUC, Department of Physics, University of Coimbra, P-3004-516 Coimbra, Portugal
[5] Institute of Geosciences, Federal University of Pará, Belém 66075-110, PA, Brazil
[6] Núcleo de Engenharia de Materiais Sustentáveis (NEMaS), Universidade do Estado do Amapá, Macapá 68900-070, AP, Brazil
[7] The Directorate of Research, Development and Innovation Management (DMCDI), Technical University of Cluj-Napoca, 15 Constantin Daicoviciu St., 400020 Cluj-Napoca, Romania
* Correspondence: stefan_ta@yahoo.com (Ş.Ţ.); nilson@academico.ufs.br (N.S.F.)

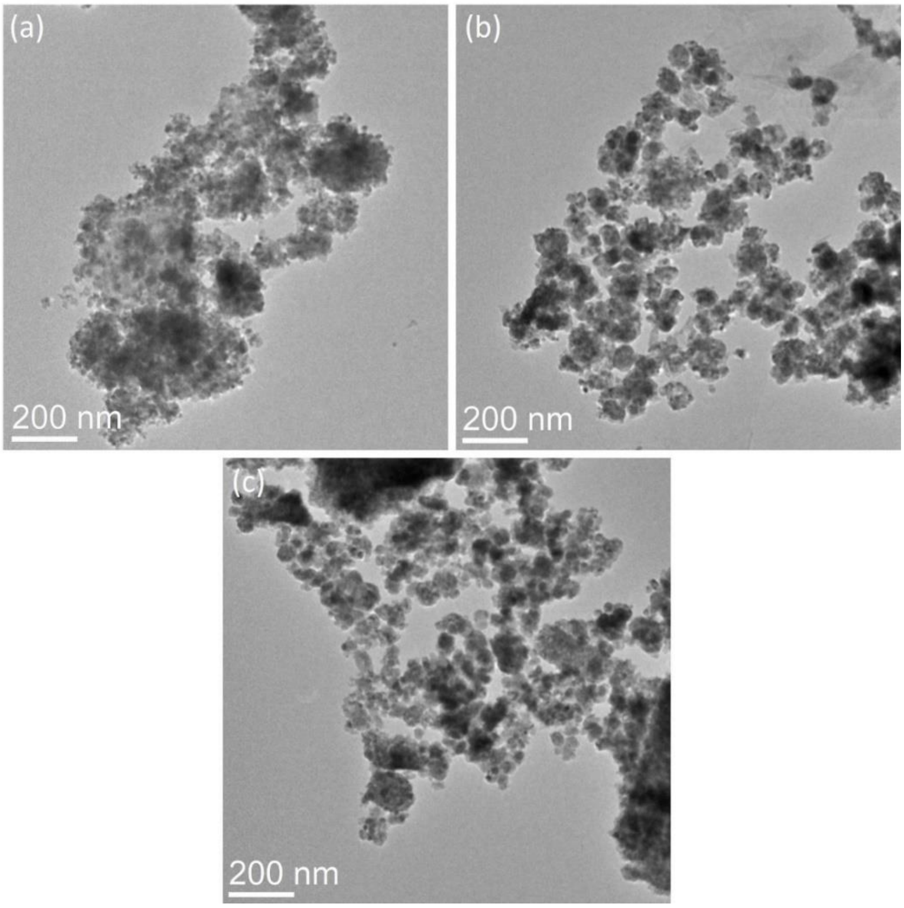

Figure S1. TEM images of (**a**) pitanga-like, (**b**) teetotum-like, and (**c**) cambuci-like ZnO nanoparticles.

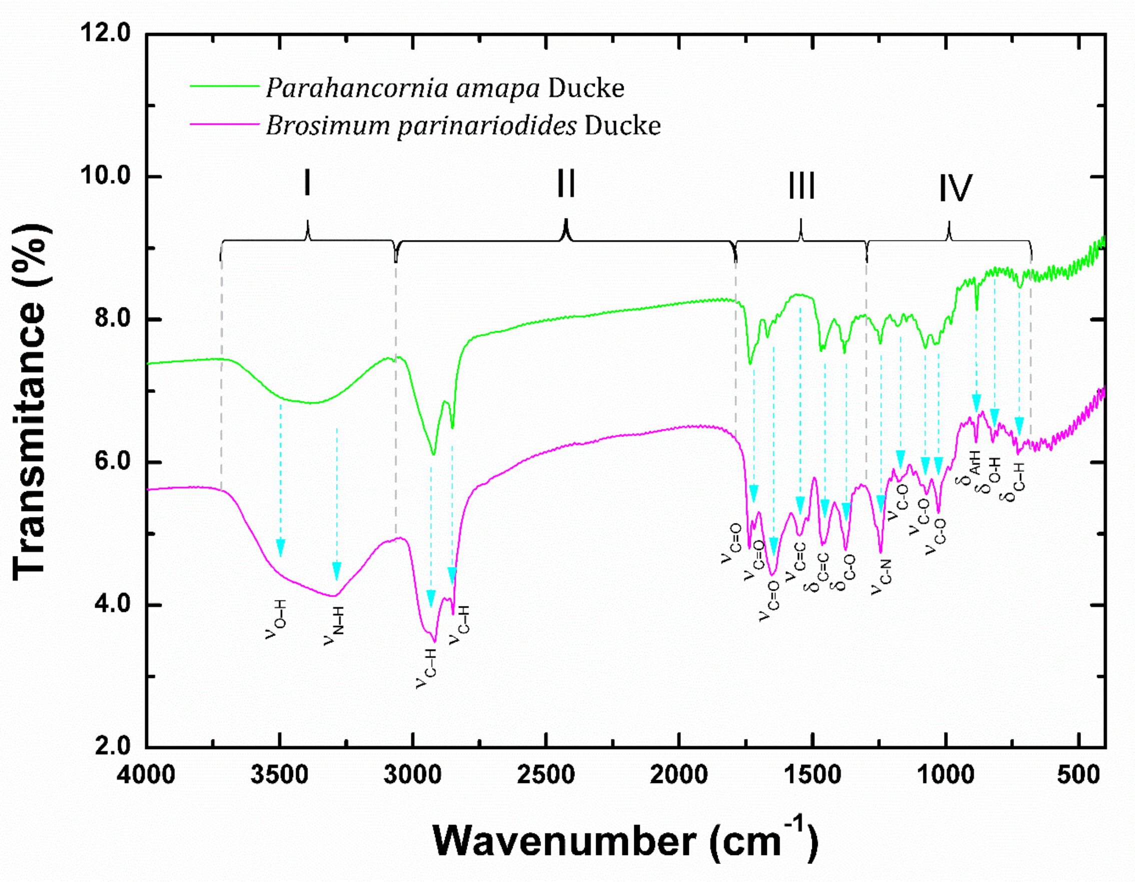

Figure S2. FTIR Spectra of freeze dried Bitter-Amapá (*Parahancornia amapa* Ducke) and Sweet-Amapá (Brosimum parinariodides Ducke) Amazon rainforest latex.
